# Electroencephalography for early Alzheimer’s disease diagnosis: from advanced feature engineering to interpretable ai and clinical translation

**DOI:** 10.3389/fpsyt.2026.1819726

**Published:** 2026-07-23

**Authors:** Mengjiao Chi, Anqi Zhao, Yixiao Zhang, Liping Fan, Qi Wang, Bing Tang, Ming Tao

**Affiliations:** 1 Zhejiang Chinese Medical University, Hangzhou, Zhejiang, China; 2 The Second Affiliated Hospital of Zhejiang Chinese Medical University, Hangzhou, Zhejiang, China

**Keywords:** Alzheimer’s disease, deep learning, early diagnosis, electroencephalogram, feature extraction, machine learning

## Abstract

**Objective:**

This review aims to comprehensively review the methodologies and advancements in using electroencephalography (EEG) for the early diagnosis of Alzheimer’s disease (AD), addressing the limitations of traditional diagnostic tools.

**Methods:**

We conducted a comprehensive analysis of current research, encompassing the complete EEG analysis pipeline from signal acquisition and preprocessing to feature extraction—including time-frequency and brain network metrics—and the application of machine learning and deep learning algorithms.

**Results:**

Characteristic EEG alterations, such as spectral slowing and reduced signal complexity, are associated with early AD. Advanced feature extraction combined with intelligent algorithms significantly enhances diagnostic performance, with some studies reporting classification accuracies exceeding 95%. Integration of EEG with multimodal data (e.g., MRI, genetic markers) further improves diagnostic robustness.

**Conclusions:**

EEG is a promising, non-invasive, and cost-effective tool for early AD detection in appropriate clinical and research settings. The integration of advanced signal processing with intelligent algorithms can significantly improves diagnostic precision, though clinical translation requires standardized protocols and validation on larger, diverse cohorts.

**Significance:**

This work highlights the potential of EEG to facilitate accessible, early-stage AD screening, which is crucial for timely intervention and may contribute to reducing the disease’s future socioeconomic burden.

## Highlights

- Provide a thorough analysis of EEG signal alterations as early functional biomarkers of AD pathology.
- Use of advanced feature extraction and machine/deep learning to enhance diagnostic accuracy.
- Discusses multimodal data integration and highlights key challenges for clinical translation, including standardization and validation gaps.

## 1 Introduction

Alzheimer’s disease (AD), a progressive neurodegenerative disorder characterized by cognitive decline and memory impairment, poses an escalating global public health challenge. With the worldwide aging population rapidly increasing, the incidence and prevalence of AD have surged correspondingly, imposing profound social, economic, and healthcare burdens. For instance, in the United States alone, an estimated 6.2 million individuals aged 65 and older were living with Alzheimer’s dementia in 2021, a figure projected to more than double by 2060 without effective interventions (1). This trend is mirrored globally, including in countries such as China, where the recent China Alzheimer Report 2025 highlights a rising incidence, prevalence, and mortality rates of AD, alongside significant economic burdens borne by families and healthcare systems (2). The disease not only affects patients but also exerts considerable strain on caregivers, who provide extensive unpaid care hours, often at the cost of their own mental and physical health (1). Moreover, disparities in diagnosis and care access persist across racial and ethnic groups, exacerbating health inequities and underscoring the need for equitable healthcare strategies (1). Although no curative therapy has yet been identified, more and more evidence indicates that early intervention can effectively slow disease progression (3, 4).

Traditional diagnostic approaches, including clinical assessment of cognitive symptoms, neuropsychological testing, and imaging modalities such as magnetic resonance imaging (MRI) and positron emission tomography (PET), still exhibit certain limitations (5, 6). The detection of amyloid-beta (Aβ) plaques and tau neurofibrillary tangles (NFTs)—key biomarkers of Alzheimer’s disease—relies on cerebrospinal fluid (CSF) analysis or neuroimaging. However, the invasiveness, high cost, and limited accessibility of these methods hinder their widespread adoption in clinical settings (5, 7). Furthermore, these methods often lack sensitivity for preclinical or prodromal AD, as neuropathological changes emerge years before overt cognitive decline (8, 9). The heterogeneity of AD pathogenesis, involving neuroinflammation, vascular factors, and genetic predispositions, further complicates early detection (10, 11). Thus, there is an imperative to develop non-invasive, cost-effective, and reliable diagnostic methods that can identify AD in its earliest stages, thereby enabling timely therapeutic interventions and potentially slowing disease progression (5, 8). Current research is increasingly focusing on novel biomarkers from peripheral biofluids, advanced neuroimaging techniques, and computational algorithms to overcome these limitations (6, 7).

With its advantages of non-invasiveness, high temporal resolution and portability, electroencephalography (EEG) has emerged as a promising neurophysiological tool for monitoring brain function and holds significant potential in the early diagnosis of AD, rendering it ideal for large-scale screening in clinical and community contexts (12). A survey on outpatient neurological examination costs reveals that EEG is over six less expensive than MRI (13). Unlike structural imaging, EEG directly measures neuronal electrical activity, providing real-time insights into functional brain alterations associated with AD pathology. Studies have demonstrated characteristic EEG changes in AD patients, such as slowing of dominant frequencies, reduced complexity, and altered connectivity patterns, which may precede clinical symptoms (12, 14). Furthermore, advanced signal processing and machine learning techniques applied to EEG data have increased the specificity of AD detection by effectively discriminating between Alzheimer’s disease, mild cognitive impairment, and healthy aging (12). These developments underscore EEG’s potential as an accessible and efficient biomarker for early AD diagnosis, complementing existing imaging and molecular methods. However, challenges remain in standardizing EEG acquisition protocols, feature extraction, and interpretation to ensure reproducibility and clinical applicability (12). Continued research integrating EEG with other diagnostic methods and leveraging intelligent algorithms is essential to fully realize its diagnostic potential in AD.

Given the critical need for early detection and the unique advantages of EEG, this review aims to comprehensively analyze current methodologies for early Alzheimer’s disease diagnosis based on brain electrical activity. We focus on recent advances in EEG signal processing, feature extraction techniques, and the application of intelligent algorithms such as hybrid optimization and machine learning to improve diagnostic accuracy. Additionally, we discuss the integration of EEG data with other biomarkers and imaging findings to enhance early diagnostic precision. Finally, we explore future trends and challenges in the field. Through this comprehensive overview, we seek to provide a foundation for advancing EEG-based early AD diagnostics, ultimately contributing to improved patient outcomes and public health strategies.

## 2 Methods for early diagnosis of AD based on EEG

### 2.1 Literature search strategy

This review adopts a narrative review approach grounded in a systematic literature search. The following electronic databases were searched: PubMed, IEEE Xplore, Scopus, and Web of Science. The search period covered January 2018 to December 2025. The search strings combined terms related to the disease, the technology, and the diagnostic methods, using the following Boolean logic: (“Alzheimer disease” OR “mild cognitive impairment”) AND (“EEG” OR “electroencephalography”) AND (“early diagnosis” OR “machine learning” OR “deep learning” OR “feature extraction”).

Studies were included if they met the following criteria: (1) original research published in English or Chinese; (2) human subjects; (3) a clear diagnosis of Alzheimer’s disease or mild cognitive impairment; (4) at least one EEG feature extraction method or machine/deep learning algorithm applied; and (5) reporting of quantitative performance metrics (e.g., accuracy, sensitivity, specificity). Exclusion criteria comprised case reports, animal studies, review articles without original data, studies with a sample size of fewer than 10 participants, and those lacking a control group.

We qualitatively evaluated study quality in the Discussion section, focusing on methodological robustness, reproducibility, and clinical relevance. The search process did not aim to exhaustively identify every publication but rather to capture the major methodological developments and representative studies in EEG−based early AD diagnosis.

### 2.2 The foundation for EEG Signal application in the early diagnosis of AD

#### 2.2.1 Physiological basis of EEG signals and their relationship with cognitive function

EEG signals originate from the summation of postsynaptic potentials generated by pyramidal neurons in the cerebral cortex, reflecting the synchronous activity of large neuronal populations. The generation mechanism involves the coordinated firing of excitatory and inhibitory neurons, producing oscillatory patterns across various frequency bands that correspond to different cognitive and behavioral states. In AD, these neural oscillations undergo characteristic alterations. Typical EEG changes in AD patients include a global slowing of the EEG spectrum, manifested as a decrease in α (8–13 Hz) and β (13–30 Hz) power, and an increase in δ(0.5–4 Hz) and θ (4–8 Hz) power, indicating disrupted cortical network dynamics (15, 16). It should be noted that such a spectral slowing pattern, while a characteristic electrophysiological feature of AD, is not a disease-specific biomarker, as it has also been observed in dementia with Lewy bodies (DLB) (17). Therefore, comprehensive assessment in differential diagnosis should integrate clinical evaluation with other biomarkers. Moreover, abnormalities in EEG rhythms, such as reduced complexity and altered synchronization, reflect the underlying synaptic dysfunction and neuronal loss. Quantitative EEG (QEEG) studies have demonstrated that the decline in cognitive functions correlates strongly with these EEG features. For instance, decreased alpha coherence and reduced global synchronization are associated with memory impairment and executive dysfunction in AD patients (18, 19). Additionally, microstate analysis reveals disorganization and reduced complexity in AD, further linking EEG patterns to cognitive decline (20). These findings underscore that EEG signals provide a window into the neurophysiological substrates of cognition, and their characteristic changes in AD can serve as early functional biomarkers of disease progression.

#### 2.2.2 EEG acquisition techniques and data preprocessing methods

Standard EEG acquisition for AD diagnosis typically employs multi-channel systems with electrode placements following the international 10–20 system, ensuring comprehensive coverage of cortical regions implicated in cognitive functions. Both conventional wet electrodes and emerging dry electrode technologies have been utilized, with dry electrodes offering advantages in ease of use and long-term monitoring, though with higher susceptibility to motion artifacts and variable skin-electrode impedance (21, 22). For long-term or ambulatory monitoring, novel approaches such as ear-EEG have been explored, demonstrating feasibility and safety in AD patients (22). Preprocessing of EEG data is critical to enhance signal quality and reliability. Common denoising techniques include bandpass filtering to remove frequency components outside the physiological range, and artifact removal methods targeting ocular (eye blinks, saccades), muscular, and cardiac interferences. Advanced algorithms such as independent component analysis (ICA) and wavelet-based filtering are employed to isolate and eliminate artifacts (23, 24). Data standardization involves normalization and segmentation into epochs to ensure consistency across analyses. These preprocessing steps are essential for accurate extraction of EEG features relevant to AD diagnosis and for minimizing confounding influences.

#### 2.2.3 Time-frequency features of EEG Signals and their significance in AD diagnosis

EEG analysis in AD leverages features extracted from the time domain, frequency domain, and combined time-frequency domain to capture the complex dynamics of brain activity. Time-domain features include statistical measures such as mean amplitude, variance, and higher-order moments like kurtosis, which reflect signal variability and complexity (25, 26). Frequency-domain analysis focuses on power spectral density (PSD) across canonical bands—δ, θ, α, and β—with AD patients exhibiting increased power in slow frequencies (δ, θ) and decreased power in faster bands (α, β), indicative of cortical slowing (15, 18). Time-frequency domain methods, such as wavelet transforms and short-time Fourier transforms, provide dynamic representations of EEG signals, enabling detection of transient oscillatory patterns and non-stationarities associated with AD pathology (27, 28). These approaches facilitate the extraction of features like event-related potentials (ERPs), spectral entropy, and connectivity metrics, which have been shown to improve diagnostic accuracy and serve as crucial biomarkers for early diagnosis and disease monitoring. For instance, using wavelet-transform-based features combined with a K-nearest neighbor classifier, Durongbhan et al. achieved a classification accuracy as high as 99% in distinguishing between Alzheimer’s disease (AD) and healthy controls on short (4 s) eyes-open EEG epochs from a cohort of 40 participants (20 AD and 20 healthy controls) (29). The integration of these features through machine learning and deep learning frameworks has further enhanced the sensitivity and specificity of EEG-based early AD detection. Gallego-Vinaras et al. applied semi-supervised deep learning to sleep-state EEG signals acquired via polysomnography, achieving 90% accuracy in its supervised form and 92–94% accuracy with fully supervised benchmarks across different sleep stages, demonstrating the feasibility of leveraging sleep EEG for early AD detection even with limited labeled data (30, 31).

### 2.3 EEG signal feature extraction methods

#### 2.3.1 Traditional feature extraction methods

Traditional EEG feature extraction methods for AD diagnosis encompass both linear and nonlinear approaches, each offering distinct insights into brain dynamics affected by AD pathology. Linear feature extraction typically involves power spectral analysis, coherence analysis, and complexity indices such as entropy measures. Power spectral analysis quantifies the distribution of EEG signal power across frequency bands (δ, θ, α, β, γ), revealing characteristic slowing of EEG rhythms in AD patients, notably increased δ and θ power and decreased α and β power (15). Coherence analysis assesses functional connectivity by measuring synchronization between EEG channels, with reduced coherence indicating disrupted neural communication in AD (32). Complexity measures like approximate entropy and sample entropy capture the irregularity and unpredictability of EEG signals, which tend to decrease in AD due to loss of neural complexity and network degradation (33). Previous studies (34), for example, Maturana-Candelas et al. (34) analyzed resting−state EEG data from 253 participants across five groups—healthy controls, mild cognitive impairment (MCI), and mild, moderate, and severe Alzheimer’s disease (AD)—and demonstrated that multiscale entropy metrics can effectively track neurodegenerative processes along the AD continuum and may serve as complementary biomarkers for monitoring disease progression.

Nonlinear feature extraction methods delve deeper into the chaotic and fractal properties of EEG signals. Fractal dimension quantifies the self-similarity and complexity of EEG time series, often reduced in AD reflecting diminished neural complexity (35). Lyapunov exponent measures sensitivity to initial conditions, with alterations indicating disrupted dynamical stability in brain activity. Sample entropy, a refined entropy measure, evaluates signal complexity and has been shown to decrease in AD, correlating with cognitive decline (36).

Biologically, these features relate closely to AD pathology (**Table 1**). The slowing of EEG rhythms and reduced coherence reflect synaptic dysfunction and neuronal loss, hallmarks of AD. Decreased complexity and fractal measures correspond to impaired neural network dynamics and reduced adaptability of brain function. Thus, traditional linear and nonlinear EEG features provide complementary biomarkers that capture both spectral and dynamical alterations induced by AD pathology, facilitating early diagnosis and monitoring of disease progression (51–53).

**Table 1:** EEG signatures as indicators of pathophysiological processes in Alzheimer’s disease: a synthesis of associations and methodological foundations.

Pathological process	Key associated EEG features	Methodological category	Main correlative findings and interpretations	Proposed physiological mechanism
Aβ Deposition (Linked to network dysfunction)	1. γ Oscillation Abnormalities (37, 38):		Abnormal γ oscillations correlate inversely with Aβ burden measured by PET, especially within DMN regions.	Aβ oligomers impair the function of inhibitory interneurons (particularly parvalbumin-positive, PV+ neurons), disrupting the network E/I balance (39). This leads to diminished high-frequency synchronization capacity and reduced efficiency of large-scale network integration.
	• Decreased power in the γ frequency band (30–100 Hz) during both rest and cognitive tasks (e.g., memory encoding).	Linear (Spectral)		
	• Weakened cross-frequency θ-γ coupling (40).	Nonlinear (Cross-Frequency Coupling)		
	2. Altered Default Mode Network (DMN) Connectivity (41): •Reduced functional connectivity in the α band (8–13 Hz), particularly within posterior cortical regions of the DMN.	Linear (Functional Connectivity)	Reduced α band connectivity strength is associated with Aβ deposition within the DMN.	
	3. Altered Background Spectral Properties (42): •Flattening of the 1/f spectral slope (most prominent in temporoparietal regions).	Nonlinear (Complexity/Fractal Analysis)	Flattened 1/f slope correlates with CSF Aβ42/40 ratio, suggesting cortical excitation/inhibition (E/I) balance disruption.	
Tau Pathology (Linked to neuronal loss and focal dysfunction)	1. Increased Slow-Wave Activity (43): •Elevated power in focal, particularly temporoparietal, δ (1–4 Hz) and θ (4–8 Hz) frequency bands.	Linear (Spectral)	The spatial distribution of slow-wave activity closely corresponds to Tau-PET signal topography.	Neurofibrillary tangles formed by tau protein lead to neuronal dysfunction and death, disrupting thalamocortical loops and long-range white matter connections. This results in focal slow-wave activity and the failure of rhythmic pacemakers.
	2. Attenuation of Physiological Rhythms (44): • Slowing of the posterior-dominant peak α frequency. • Widespread reduction in α and β (13–30 Hz) power.	Linear (Spectral)	Slowing of α frequency shows a significant negative correlation with medial temporal lobe tau burden.	
	3. Reduced Network Efficiency (45, 46): • Decreased global and local efficiency of functional networks based on graph theory analysis.	Nonlinear (Network Science)	Declining network efficiency correlates with tau-mediated structural disconnection and cortical atrophy.	
Synaptic Loss/Dysfunction (The direct structural substrate of cognitive decline)	1. Attenuated Event-Related Potentials (ERPs): • Increased latency and decreased amplitude of the P300 (P3b) component (47). • Reduced amplitude of the Mismatch Negativity (MMN) (48, 49).	Linear (Time-Domain Averaging)	Reduced P300 amplitude emerges early in the disease course and is a sensitive indicator of diminished cognitive processing resources, closely related to synaptic density.	The loss of pre-synaptic terminals and post-synaptic structures directly reduces the size of the neuronal pool available for synchronized firing and undermines the neural substrate for rapid information transfer.
	2. Generalized Suppression of High-Frequency Oscillations (50): • Widespread decrease in resting-state β and γ band power.	Linear (Spectral)	High-frequency oscillation power is considered a real-time “electrophysiological readout” of synaptic efficacy and neuronal population synchronizability.	

EEG, electroencephalography; DMN, default mode network; PET, positron emission tomography; CSF, cerebrospinal fluid; E/I, excitation/inhibition; MMN, mismatch negativity; ERPs, event-related potentials.; MRI, Magnetic resonance imaging; PET, Positron emission tomography.

#### 2.3.2 Advanced feature extraction techniques

Traditional feature extraction methods establish an interpretable framework of physiological mechanisms for understanding EEG abnormalities in AD, whereas advanced feature extraction techniques build upon this foundation to deliver enhanced discriminative power with greater dimensionality and sensitivity through data-driven pattern mining. These advanced techniques primarily encompass three key methodological pillars that enable sophisticated pattern discovery while enhancing the sensitivity and specificity of AD diagnosis: time-frequency analysis, brain network modeling, and multimodal data integration.

Time-frequency joint analysis methods, such as wavelet transforms (54) and short-time Fourier transforms (55), enable simultaneous examination of temporal and spectral EEG characteristics, capturing transient changes and non-stationarities inherent in AD-related brain activity. These methods overcome limitations of purely time- or frequency-domain analyses by revealing dynamic oscillatory patterns and their evolution, which are crucial for detecting subtle AD-associated alterations.

Brain network analysis, grounded in graph theory, models the brain as a complex network where nodes represent EEG channels or brain regions and edges denote functional connectivity (56). Metrics such as clustering coefficient, path length, modularity, and small-worldness quantify network integration and segregation. In AD, these network properties are disrupted, reflecting impaired communication and altered topology (57). Functional connectivity measures derived from coherence or phase synchronization form the basis for constructing these networks, enabling identification of network hubs and patterns predictive of AD.

Multimodal fusion integrates EEG features with other biomarkers, such as structural MRI (58), functional MRI (59), or cerebrospinal fluid markers (60), to exploit complementary information. Feature-level fusion combines heterogeneous data into a unified representation, enhancing classification performance. For example, combining EEG-derived functional connectivity with MRI-based structural features improves discrimination between AD, mild cognitive impairment, and healthy controls (61). This integrative approach addresses the multifactorial nature of AD and leverages diverse biological signatures for robust diagnosis.

#### 2.3.3 Feature selection and dimensionality reduction

Feature selection and dimensionality reduction are critical for optimizing EEG-based AD diagnostic models by enhancing classification accuracy, reducing computational burden, and improving interpretability. Three main categories of feature selection methods are employed: filter, wrapper, and embedded approaches. Filter methods evaluate features independently of classifiers using statistical metrics such as correlation, mutual information, or F-statistics, offering computational efficiency but ignoring feature dependencies. Wrapper methods assess subsets of features by training classifiers, providing higher accuracy at the expense of increased computational cost. Embedded methods integrate feature selection within model training, such as LASSO or tree-based algorithms, balancing performance and efficiency.

Dimensionality reduction techniques like Principal Component Analysis (PCA) and Linear Discriminant Analysis (LDA) transform high-dimensional EEG features into lower-dimensional spaces (62). PCA identifies orthogonal components capturing maximum variance without considering class labels, useful for noise reduction and visualization. LDA seeks projections maximizing class separability, enhancing discriminative power for classification tasks. These methods mitigate the curse of dimensionality and overfitting, common in EEG data with numerous correlated features.

Empirical studies demonstrate that appropriate feature selection and dimensionality reduction substantially improve diagnostic performance. For instance, combining maximal relevance(mRMR) filter selection with Support Vector Machine (SVM) classifiers enhances accuracy in AD classification (63, 64). Adaptive stepwise selection and evolutionary algorithms have also been applied to identify optimal feature subsets, yielding robust models (65). Moreover, feature selection contributes to model interpretability by highlighting biologically relevant EEG features linked to AD pathology. Overall, tailored feature selection and dimensionality reduction strategies are indispensable for developing effective EEG-based AD diagnostic systems (66–68).

### 2.4 Intelligent algorithms for early AD diagnosis based on EEG

**Figure 1:**
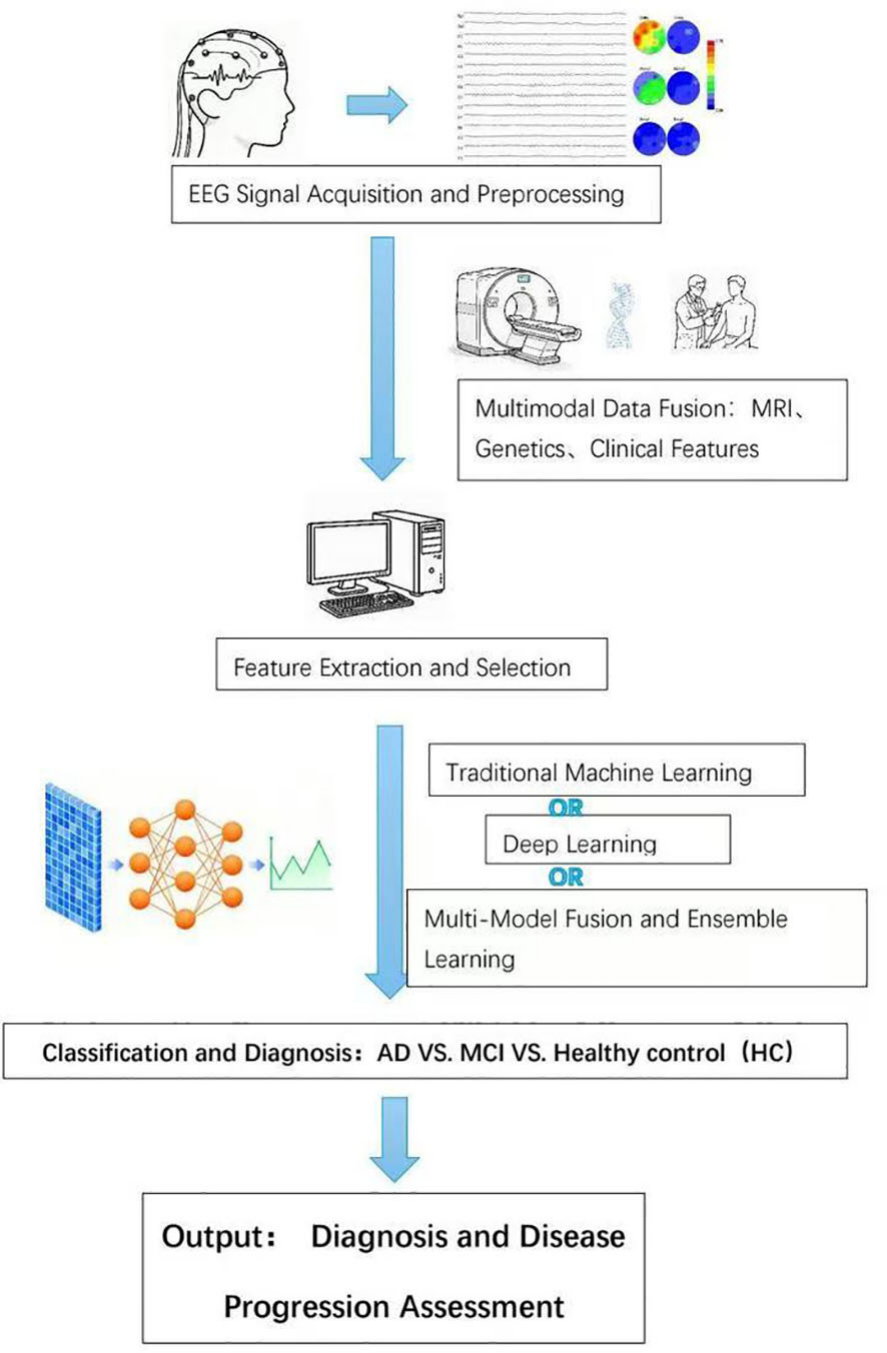
Technical roadmap for EEG-based early diagnosis of AD. EEG, electroencephalography; MRI, Magnetic resonance imaging. This flowchart systematically illustrates the pipeline from raw EEG acquisition to diagnostic output, encompassing critical steps of data preprocessing, feature engineering, model construction, and multimodal integration.

Before applying any algorithm, feature selection and dimensionality reduction must be performed (**Figure 1**), as this is a foundational step for enhancing model performance and preventing overfitting. Notably, current research exhibits a distinct transition from traditional machine learning, which relies on such handcrafted features, toward deep learning models capable of automated feature extraction.

#### 2.4.1 Traditional machine learning algorithms

Traditional machine learning algorithms such as SVM, Random Forest (RF), and k-Nearest Neighbors (k-NN) have been extensively applied to EEG data for early diagnosis of Alzheimer’s disease (AD). SVM, known for its strong generalization ability in high-dimensional spaces, has demonstrated high classification accuracy in differentiating AD patients from healthy controls and other dementia types. Senkaya et al. (2025) employed a machine learning approach to analyze electroencephalography (EEG) data from 36 patients with Alzheimer’s disease (AD), 23 patients with frontotemporal dementia (FTD), and 29 healthy controls. A total of 342 statistical and spectral features were extracted from time-domain signals and power spectral density (PSD). Feature selection was performed using correlation analysis, followed by classification using a support vector machine (SVM) with hyperparameters optimized via Bayesian optimization. The SVM classifier achieved an accuracy of 96.01% (69). Similarly, RF, an ensemble learning method based on decision trees, has shown robust performance by effectively handling the nonlinear and noisy nature of EEG signals. Comparative studies indicate that RF can achieve competitive accuracy while providing feature importance insights, which are valuable for understanding AD-related EEG biomarkers (70). Lal et al. (2024) employed a sliding window technique with 90% overlap, extracted features using singular value decomposition (SVD) entropy, and applied a k−nearest neighbors (k−NN) classifier on EEG data from 88 subjects (36 Alzheimer’s disease, 23 frontotemporal dementia, and 29 healthy controls). The model achieved classification accuracies exceeding 90%: 94.72% for discriminating AD from healthy controls and 94.18% for discriminating FTD from healthy controls (71). The comparative evaluation of these algorithms often reveals trade-offs between classification accuracy and computational complexity. SVM tends to provide superior accuracy and generalization but may require careful kernel and parameter tuning, while RF offers interpretability and resistance to overfitting. k-NN, although straightforward, may suffer in scalability and sensitivity to noisy data. Building effective training datasets involves careful preprocessing, including artifact rejection and feature extraction such as power spectral density, entropy, and connectivity measures. Cross-validation strategies, predominantly k-fold and leave-one-subject-out methods, are employed to assess model generalization and prevent overfitting (72, 73). These traditional ML approaches provide a solid foundation for EEG-based AD diagnosis, yet their reliance on handcrafted features and limited capacity to capture complex temporal dynamics motivates the exploration of deep learning models.

#### 2.4.2 Deep learning models and their advantages

Deep learning models, particularly convolutional neural networks (CNNs) and recurrent neural networks (RNNs) including long short-term memory (LSTM) networks, have revolutionized EEG analysis for early AD diagnosis by enabling automatic feature extraction and modeling of temporal dependencies. CNNs excel in capturing spatial and spectral EEG features through hierarchical convolutional layers, effectively learning discriminative representations from raw or time-frequency transformed EEG data. For example, CNN-based models trained on EEG time-frequency images achieved classification accuracies around 83-88% for differentiating AD and mild cognitive impairment (MCI) from healthy controls, demonstrating superior performance over traditional ML methods (74). RNNs and LSTMs are particularly adept at handling sequential EEG data, capturing temporal patterns critical for AD diagnosis. Attention-based LSTM models have shown enhanced ability to focus on salient temporal features, improving classification accuracy in task-based EEG paradigms (75). End-to-end deep learning frameworks further integrate feature extraction and classification, streamlining the diagnostic pipeline. Alouani et al. (2025) developed a contrastive dual−branch network (CDB−Net) trained on 88 subjects (36 AD, 23 FTD, 29 HC) that jointly learns from clean and noisy EEG signals, achieving 97.92% accuracy on clean data and 83.41% accuracy under FGSM adversarial attacks (76). Moreover, hybrid architectures combining CNNs with LSTM or attention mechanisms have demonstrated the capacity to fuse spatial and temporal EEG features effectively, leading to improved diagnostic precision (77). These deep learning models reduce the dependency on manual feature engineering and can generalize better across subjects and recording conditions. However, challenges remain in requiring large labeled datasets and addressing inter-subject variability. Transfer learning and multi-task learning strategies have been proposed to mitigate data scarcity and enhance model generalization (78, 79). Overall, deep learning approaches represent a promising direction for automated, accurate, and scalable EEG-based early AD diagnosis.

#### 2.4.3 Multi-model fusion and ensemble learning

To further enhance the accuracy and robustness of EEG-based early AD diagnosis, multi-model fusion and ensemble learning strategies have been increasingly adopted. Multi-model fusion involves combining predictions or features from multiple classifiers or models to leverage their complementary strengths. For example, integrating outputs from SVM, RF, and k-NN classifiers through weighted voting or stacking has been shown to improve classification performance beyond individual models (80). Ensemble learning methods such as Boosting and Bagging have also been applied to EEG data classification. Boosting algorithms iteratively focus on misclassified samples to build strong classifiers, while Bagging reduces variance by training multiple models on bootstrapped datasets. These approaches enhance model stability and generalization, particularly in the presence of noisy EEG signals (81). Beyond classifier-level fusion, multi-source information integration combining EEG features with clinical data (e.g., neuropsychological assessments, genetic information) has demonstrated superior diagnostic accuracy. Yu et al. (2024) trained an SVM model on EEG and genetic features (SNPs and PRS) from 60 AD patients and 83 controls, achieving a cross−validation accuracy of 92.1% with multimodal data, which significantly outperformed single−modality models and demonstrated the value of data fusion (70). Additionally, frameworks employing decision-level fusion of EEG-derived biomarkers and imaging or cognitive scores have been proposed to provide comprehensive diagnostic insights (82).These multi-model and multimodal fusion strategies address limitations of single-model approaches by capturing diverse aspects of AD pathology and enhancing robustness against inter-subject variability and recording artifacts. However, challenges such as optimal fusion strategy selection, computational complexity, and data harmonization remain active research areas. Future work focusing on adaptive, interpretable ensemble models and integration of heterogeneous data sources holds promise for advancing EEG-assisted early AD diagnosis.

#### 2.4.4 Interpretability and explainable AI in EEG-based AD diagnosis

Despite the remarkable accuracy achieved by deep learning models in EEG-based AD classification, their clinical adoption remains severely limited by the “black-box” nature. Clinicians cannot trust a diagnostic suggestion without understanding why the model made a particular decision, especially in high-stakes neurodegenerative disease diagnosis (83). Explainable artificial intelligence (XAI) addresses this critical gap by providing human−understandable rationales behind model predictions, thereby fostering trust, enabling model validation, and potentially discovering novel electrophysiological biomarkers.

XAI methods can be broadly categorized into intrinsic interpretable models and post−hoc explanation methods. Intrinsic models (e.g., logistic regression, decision trees, and explainable boosting machines, EBM)) are inherently transparent but often underperform on complex EEG data. Post−hoc methods, which are the mainstream in EEG−AD research, are applied after training a black−box model (e.g., deep neural networks) without altering its parameters. These methods attempt to explain specific predictions made by the already−trained complex model. While flexible, post−hoc explanations are only approximations of the model’s internal logic and carry the risk of incomplete faithfulness to the actual decision−making process. They can be further divided into model−specific vs. model−agnostic and local vs. global explanations.

Several XAI techniques have been successfully applied to EEG−AD diagnosis as showed in **Table 2**. Despite these advances, several challenges remain. Faithfulness (whether the explanation truly reflects the model’s internal logic) and robustness (stability under small input perturbations) are difficult to quantify. Different XAI methods often produce divergent explanations for the same prediction, raising consistency concerns. Moreover, standardized benchmarks and evaluation protocols for XAI in EEG−AD are lacking; most assessments still rely on visual inspection. Finally, computational overhead—especially for perturbation−based methods like SHAP—may hinder real−time deployment in clinical settings. Future directions should focus on developing causally−grounded explanations and interactive interfaces that allow clinicians to query the model in a human−in−the− loop manner. Establishing publicly available benchmarks with ground−truth explanations (e.g., simulated EEG lesions or simultaneous fMRI−EEG data) and standardised faithfulness metrics will be crucial for translating XAI−enhanced EEG−AD models from research laboratories into routine clinical practice.

**Table 2:** Summary of explainable AI (XAI) techniques applied to EEG−based Alzheimer’s disease diagnosis.

XAI technique	Core principle	EEG−AD application example EEG-AD	Main advantages	Limitations	Main references
Local Interpretable Model-agnostic Explanations (LIME)	Locally approximates a black−box model with an interpretable surrogate model (e.g., linear regression) by generating perturbed samples.	Identifies which EEG channels, time segments, or frequency bins most influence a single AD prediction.	Model−agnostic; provides local explanations; easy to implement.	Unstable (different perturbations may yield different explanations); computationally expensive for high−dimensional EEG data.	Tokdar et al,2026 (84); Atf et al,2025 (85)
SHapley Additive exPlanations (SHAP)	Based on Shapley values from cooperative game theory; fairly distributes the prediction difference from the baseline among input features.	Reveals global feature importance (e.g., δ/θ power, posterior α slowing) and local contributions per sample; validates that the model learned biologically meaningful patterns.	Model−agnostic; both local and global explanations; solid theoretical foundation.	Computationally intensive, especially for high−dimensional or raw EEG signals; approximation methods (e.g., KernelSHAP) may trade off accuracy.	Khan et al,2025 (86); Atf, et al,2025 (85); Tokdar et al,2026 (84)
Saliency Maps	Computes the gradient of the output class score with respect to the input; highlights input features with large gradients.	Generates sensitivity maps over raw EEG time series or spectrograms, indicating which time–frequency points drive the AD classification.	Fast, easy to compute for differentiable models (CNNs, LSTMs).	Often noisy; suffers from gradient saturation and may not faithfully reflect model logic.	Lopes et al, 2023 (87); Sun et al, 2025 (88)
Gradient-weighted Class Activation Mapping (Grad−CAM)	Uses gradients flowing into the last convolutional layer to produce a coarse heatmap localizing important regions in the input image (e.g., spectrogram).	When EEG is converted to spectrograms or topographic maps, Grad−CAM visually highlights which frequency–time patches (e.g., persistent theta over temporal areas) contributed most to an AD diagnosis.	Intuitive heatmap visualization; better localization than simple saliency maps; widely used for CNN−based EEG analysis.	Low spatial resolution (depends on last conv layer); only applicable to CNNs with image−like inputs.	Morabito et al,2023 (89); Abdulaal et al, 2026 (90); Bravo-Ortiz et al, 2024 (91)
Attention Visualization	Utilizes the attention weights from Transformer or attention−augmented RNNs as intrinsic explanations.	Visualizes which time points, channels, or cross−channel interactions the model prioritizes (e.g., higher attention to parieto−occipital electrodes during 300−500 ms post−stimulus for P300 analysis).	No extra computation; part of the model architecture; can reveal temporal and spatial dependencies.	Attention weights do not always faithfully represent causal importance; may be misleading.	Bravo-Ortiz et al, 2024 (91); Khosravi et al, 2024 (92); Abdulaal et al, 2026 (90)

## 3 Discussion

### 3.1 Consistent EEG alterations and confounding factors in Alzheimer’s disease

Despite the consistent EEG signatures of AD—namely spectral slowing (increased δ/θ power, decreased α/β power), reduced functional connectivity in the α band, and prolonged P300 latency—substantial inter−study variability persists due to three categories of confounding factors. Patient−related factors include age (normal aging mimics mild slowing), comorbidities (cerebrovascular disease, depression, sleep apnea), and medications (93, 94), particularly cholinesterase inhibitors which partially normalize the EEG spectrum and systematically underestimate disease−control differences (95). Study design factors involve heterogeneous diagnostic criteria, disease stage (MCI vs. moderate AD), and control group selection (possible preclinical pathology). Technical factors—acquisition hardware, electrode montage, recording conditions, and preprocessing pipelines (filtering, artifact rejection, re−referencing)—constitute the most pervasive yet under−reported source of variability, leading to the reproducibility crisis. Recognizing these, standardization initiatives such as Brain Imaging Data Structure for Electroencephalography (BIDS−EEG), Standardized Early−stage EEG Processing Pipeline (PREP), and ComBat harmonization are urgently needed to move EEG−based AD biomarkers toward clinical acceptance.

### 3.2 Barriers to clinical translation of EEG− based AD diagnosis

Despite the promising advances in feature extraction and intelligent algorithms reviewed above, the translation of EEG−based early AD diagnosis into routine clinical practice remains severely limited. As of 2026, no dedicated quantitative EEG (qEEG) software for AD screening has received regulatory approval (e.g., FDA or EMA), and clinical practice guidelines largely overlook EEG as a diagnostic biomarker (96).

#### 3.2.1 Methodological and validation gaps

First, lack of standardization across the entire EEG pipeline—from acquisition protocols (electrode montage, reference, sampling rate) to preprocessing (filtering, artifact rejection) and feature extraction—severely hampers reproducibility and cross−study comparabilit. Unlike neuroimaging (e.g., ADNI harmonized protocols) or fluid biomarkers, no widely accepted international standard exists for qEEG in AD research. This “analytical flexibility” inflates performance estimates and undermines trust. Second, most studies rely on small, single−center, convenience samples with narrow demographic and clinical characteristics. Models that achieve satisfyingly high accuracy in such curated datasets often fail to generalize to real−world populations with comorbidities, medication use, or racial/ethnic diversity. Third, the vast majority of published work is cross−sectional, comparing established AD patients to healthy elderly. Longitudinal evidence demonstrating that EEG features predict conversion from mild cognitive impairment (MCI) to AD—the key requirement for an early diagnostic tool—remains scarce. Large, prospective, multi−center cohort studies are urgently needed to establish predictive validity.

#### 3.2.2 Regulatory hurdles for AI−based software as a medical device

Even when a model demonstrates robust performance, navigating the regulatory landscape is daunting. Traditional approval pathways (e.g., FDA 510(k)) rely on “substantial equivalence” to a predicate device; however, no such predicate exists for deep learning−based qEED AD classifiers. The “black−box” nature of deep neural networks complicates safety and effectiveness evaluation, and adaptive algorithms that continuously learn post−deployment fall outside current locked−algorithm frameworks (86). Importantly, neither the FDA nor the EMA has issued specific guidance on the required sensitivity/specificity thresholds for EEG−based cognitive screening devices, nor detailed validation protocols tailored to qEEG. The absence of a clear regulatory pathway, combined with the high cost of pivotal clinical trials, has resulted in zero approved products for EEG−based AD diagnosis as of mid−2026.

#### 3.2.3 Practical obstacles in primary care

Even if regulatory approval and guideline endorsement were achieved, deploying EEG−based screening in primary care—the intended point−of−care—faces substantial logistical challenges. Traditional wet−electrode systems require shielded rooms and trained technicians; while dry−electrode portable devices exist, their signal quality and usability in older adults need real−world validation. Primary care physicians lack training in EEG interpretation, and adding a screening step would disrupt already overloaded workflows.

#### 3.2.4 Towards a roadmap for translation

Overcoming these barriers will require coordinated efforts across stakeholders. Academic and industry consortia should prioritize the development of standardized acquisition and preprocessing protocols (similar to the Alzheimer’s Disease Neuroimaging Initiative for MRI). Large, multi−center, prospective longitudinal studies (e.g., thousands of participants with PET or CSF confirmation) must be funded to establish predictive validity and clinical utility. Regulators need to create tailored guidance for AI−based qEEG devices, including acceptance criteria for analytical and clinical validation. Guideline committees should periodically reassess evidence as it emerges, potentially endorsing EEG as a low−cost triage tool to enrich high−risk populations for confirmatory testing. Finally, health economists and payers must collaborate to generate cost−effectiveness data that can inform coverage decisions. Only through such systemic, multi−disciplinary efforts can the translational chasm be bridged, transforming EEG from a promising research tool into a clinically actionable early diagnostic solution for Alzheimer’s disease.

## 4 Conclusion

EEG offers a non−invasive, cost−effective, and temporally precise window into early Alzheimer’s disease pathophysiology, capturing functional alterations such as spectral slowing and network disruption before overt clinical symptoms. When combined with advanced feature extraction and machine learning—including deep learning, multimodal fusion, and explainable AI—EEG−based models have achieved high diagnostic accuracy in research settings.

However, translation to clinical practice remains hindered by methodological heterogeneity, confounding factors (age, medications, comorbidities), lack of standardization, and the absence of regulatory-approved products or guideline endorsement. Overcoming these barriers requires large−scale, multi−center longitudinal studies, adoption of standardized pipelines (e.g., BIDS−EEG, PREP, ComBat), and coordinated efforts among researchers, clinicians, regulators, and payers. With such systemic progress, EEG can evolve from a promising research tool into a reliable, accessible early diagnostic solution for Alzheimer’s disease, enabling timely intervention and improved patient outcomes.
